# Efficacy and safety of recruitment maneuvers in acute respiratory distress syndrome

**DOI:** 10.1186/2110-5820-1-9

**Published:** 2011-04-19

**Authors:** Claude Guerin, Sophie Debord, Véronique Leray, Bertrand Delannoy, Frédérique Bayle, Gael Bourdin, Jean-Christophe Richard

**Affiliations:** 1 Service de Réanimation Médicale, Hôpital de la Croix-Rousse, 103 Grande Rue de la Croix-Rousse, Lyon, 69004 France

## Abstract

Recruitment maneuvers (RM) consist of a ventilatory strategy that increases the transpulmonary pressure transiently to reopen the recruitable lung units in acute respiratory distress syndrome (ARDS). The rationales to use RM in ARDS are that there is a massive loss of aerated lung and that once the end-inspiratory pressure surpasses the regional critical opening pressure of the lung units, those units are likely to reopen. There are different methods to perform RM when using the conventional ICU ventilator. The three RM methods that are mostly used and investigated are sighs, sustained inflation, and extended sigh. There is no standardization of any of the above RM. Meta-analysis recommended not to use RM in routine in stable ARDS patients but to run them in case of life-threatening hypoxemia. There are some concerns regarding the safety of RM in terms of hemodynamics preservation and lung injury as well. The rapid rising in pressure can be a factor that explains the potential harmful effects of the RM. In this review, we describe the balance between the beneficial effects and the harmful consequences of RM. Recent animal studies are discussed.

## Definition

Recruitment maneuvers (RM) can be defined as a voluntary strategy to increase the transpulmonary pressure (P_L_) transiently with the goal to reopen those alveolar units that are not aerated or poorly aerated but reopenable. The consequence of this should be the induction of lung recruitment. This strategy can be performed by using the conventional ICU ventilator or the high-frequency oscillation device in the supine or prone positions. This review concentrates on the MR performed with the conventional ICU ventilators in the supine position.

## Rationale

The rationale of using RM in patients with the acute respiratory distress syndrome (ARDS) stems from three considerations.

### 1. ARDS lung is derecruited and recruitable

The loss of aerated lung volume is the cardinal feature of ARDS as demonstrated by numerous studies that used lung computed tomography (CT) scan [1-3]. Alveolar collapse (i.e., atelectasis) results from increased interstitial pressure and weight of the lung (sponge theory). It can be enhanced by patient-related factors, such as obesity, increased intra-abdominal pressure, high levels of inspired oxygen in unstable alveoli, patient disconnection from the ventilator, or tracheal suctioning. It should be stressed that by definition ARDS is a lung permeability edema, which means that alveoli are not collapsed, i.e., airless, but liquid-filled. Alveoli also can be filled by inflammatory cells or blood.

The lung in ARDS can be reaerated by increasing P_L_, or more exactly transalveolar pressure (= alveolar pressure minus interstitial pressure). The amount of lung mass that can be recruited, named the lung recruitability, has been found to be quite low, averaging 9% of the total lung mass, between 5 and 45 cm H_2_O [4]. Other investigators have found, by contrast, that all of the lung mass can be reopened in early ARDS if a sufficient amount of P_L _is generated to go over the critical opening pressure (COP) of the lung units [5,6].

### 2. Concept of COP of the lung units

According to this concept, the closed terminal respiratory units should reopen once a minimal amount of regional P_L _to maintain patency of small airways and/or alveoli has been reached. Depending on the mechanisms and location of closure of the terminal respiratory units, the amount of COP should vary from relatively low values, such 10 cm H_2_O, to very high values. In humans, COP values have been found to follow a Gaussian distribution with a mode of approximately 25 cm H_2_O [7] or a bimodal distribution with a second mode close to 40 cm H_2_O [5]. It must be stressed that the full range of regional COP was as wide as 0 to 60 cm H_2_O [5,7].

### 3. Lung recruitment is beneficial

Recruiting the lung is a ventilatory strategy that can prevent ventilator-induced lung injury (VILI) [8]. This benefit may result from two mechanisms. The first is the increase in the aerated lung mass, which contributes to minimize the lung heterogeneity and to increase the size of the baby lung. The second is the prevention of the repeated opening and closure of the terminal respiratory units.

**Figure 1:**
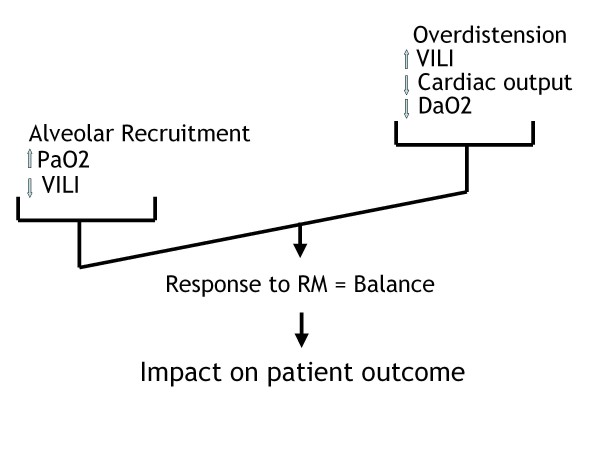
**Balance between benefits (left tray) and risks (right tray) of the recruitment maneuvers**. VILI, ventilator-induced lung injury; RM, recruitment maneuver; DaO2, oxygen transport.

RMs have probably long been used mostly to improve oxygenation, which is a good thing if this improvement results from or is associated with lung recruitment. However, the global effect of RM is actually a balance between positive effects (reduction in VILI, improvement in oxygenation) and negative effects (increase in VILI, hemodynamics impairment). From this balance, one can expect favorable or poor outcome of the patient (Figure 1).

## Methods to recruit the lung

The RMs are not unique, which is a general limitation of the technique because it is not standardized as yet. The earliest RM ever used during mechanical ventilation is probably the sigh [9], which consists of increasing tidal volume or level of positive end-expiratory pressure (PEEP), depending on the ventilator used, for one or several breaths. Tidal volume and PEEP level can be adjusted to reach a specific plateau pressure (Pplat). Pelosi et al. [10] in ten patients with ARDS applied three consecutive sighs per minute, each of them generating Pplat of 45 cm H_2_O, and found that oxygenation was better, lung static elastance lower, and functional residual capacity (FRC) greater in the 1-hour-sigh period than in the no-sigh period. However, some safety concern could have been raised given that this schedule would lead to 4,320 occurrences per day of Pplat 45 cmH_2_O, which is a level well above the 30 cm H_2_O recommended threshold to maintain in ARDS [11]. The most frequently investigated RM, due to its apparent simplicity, is the sustained inflation (SI), which consists of pressurizing the airways at a specific level and maintaining it for a given duration. A common combination is the application of 40 cmH_2_O airway pressure for 40 seconds [12-14]. In a randomized controlled trial involving 30 patients with ARDS, SI of 50 cmH_2_O applied for 30 seconds did not result in better oxygenation by 30 minutes compared with the control group free of RM [13]. In that study, SI was applied after PEEP had been standardized in both groups similarly. The interaction between pressure and time is critical in the efficacy and tolerance of RM. Therefore, some authors introduced the extended sigh [15], which combines lower pressure level, progressive rising of airway pressurization, and longer time of application. High PEEP and pressure-controlled ventilation with a fixed driving pressure (= inspiratory pressure minus PEEP) are other ways to perform RM [5].

The RMs were compared each other in some investigations. It should be stressed that an adequate comparison is difficult due to the pressure-time product, which should be made identical between the two RMs. For example, in 19 patients with ARDS, extended sigh was associated with better oxygenation and higher recruited volume than single SI 40 cm H_2_O for 40 seconds [16]. Using two or more RMs would have led to different results. We compared optimal PEEP alone, selected from a decremental PEEP trial, SI + optimal PEEP and sighs + optimal PEEP in 12 patients with ARDS in a cross-over study and found that sighs were associated with better oxygenation and greater static compliance of the respiratory system than any other strategy [17].

The meta-analysis of the studies on RMs in ALI/ARDS by Fan et al. [18] concluded that RMs were neither recommended nor forbidden and could rather be used on a case-by-case basis in the most hypoxemic patients as a life-saving procedure. Another systematic review did not recommend the systematic use of RMs in the routine practice in "stable" ARDS patients [19]. It should be mentioned that, apart from severely hypoxemic ARDS patients where RMs could be used to maintain safe oxygenation levels, RMs should be applied after tracheal suctioning [20] or patient disconnection. In the early trial, which introduced the concept of lung protective mechanical ventilation [21], RMs were managed after tracheal suctioning.

Four lines of considerations cast some doubt about the routine use of RMs in patients with ARDS.

1. The fact that three randomized, controlled trials were not able to demonstrate a beneficial effect of RMs on oxygenation in the routine practice [13,22,23].

2. Some safety concerns [24].

3. The large variability of the oxygenation response across the patients [23].

4. The relevant end-points in the assessment of RMs have moved from the oxygenation improvement toward the VILI prevention.

## Factors of the response to RMs

As shown in Table 1, several factors are involved in the response to RMs in terms of oxygenation, lung recruitment, or hemodynamics. Some of these effects are discussed below.

**Table 1:** Factors potentially involved in the variability of the response to recruitment maneuvers in ARDS

**ARDS-related**	
	Focal vs. nonfocal
	Early vs. Late
	Severe vs. moderate
	Lung recruitability
	Associated vasoactive drugs
**RM-related**	
	Type of recruitment maneuvers
	Distribution of lung perfusion
	Transpulmonary pressure
	Timing of application
	Patient positioning
**Post-RM strategy**	
	Post-RM PEEP

ARDS, acute respiratory distress syndrome; RM, recruitment maneuvers; PEEP, positive ned-expiratory pressure.

### Type of ARDS

This is a major factor because ARDS is highly heterogeneous by nature, both within patients and between patients. The separation between focal and not focal ARDS has been largely accepted. Constantin et al. [25] separated ARDS patients into focal and not focal morphological patterns from the CT scan and studied the effect of a single SI applied before and after open lung ventilation, namely a high PEEP. After the RM, the oxygenation remained unchanged in the focal whilst it improved in the not focal ARDS pattern. Most importantly, in the focal pattern after the RM, the lung overdistension markedly increased and was greater than the lung recruitment elicited by RM. Once RM was released, the overdistension remained above its level before RM. In sharp contrast in the not focal ARDS pattern, the recruited volume markedly increased and was greater than the concomitant overdistension with the RM. After the RM, the overdistension went back to its baseline level but the recruited volume remained higher than its pre-RM level. This result was extended by Grasso et al. [26] who investigated the effect of a single SI in three experimental ARDS in pigs: surfactant depletion with massive derecruitment and no inflammation; oleic acid-induced ARDS with massive lung edema and no inflammation; and hydrochloride acid-induced ARDS characterized by massive inflammation. The RM did promote recruitment but also overdistension in the most anterior parts of the lungs in the three ARDS models, making the lungs more heterogeneous than before the RM application. Furthermore, the overdistension, and hence the lung heterogeneity, was maintained after RM release. The morphological lung heterogeneity was associated with a marked functional heterogeneity because the elastance of the recruited parts of the lungs was significantly greater than in the control animals and than that of the baby lung in each ARDS model. This result is very important to keep in mind when RM is used.

Another criterion to separate ARDS patients is the severity of the lung injury. Whereas it is difficult to accurately and precisely define what severe ARDS is, the paraquat model of ARDS in rats is useful in this purpose because the lung histomorphometry findings are different with the dose of paraquat administered. The intraperitoneal injection of 20 mg/kg paraquat induces alveolar collapse and interstitial oedema whilst a greater dose of 25 mg/kg promotes an additional alveolar oedema. Therefore, low dose of paraquat induced moderate ARDS whilst with high dose of paraquat severe ARDS would follow. Santiago et al. [27] found that a single SI induced a significantly greater magnitude of overdistension, endothelial and epithelial alveolar cells injury, and apoptosis to the lungs and kidneys in severe than in moderate paraquat-induced ARDS in rats.

### Lung perfusion

Lung perfusion is a critical determinant of oxygenation. In a sheep model of surfactant depletion, a single SI worsened oxygenation in every animal [28]. The mechanism of this finding was that: 1) the RM did not recruit the dorsal part of the lungs in which there was a massive loss of aeration, and 2) redistributed the pulmonary blood flow toward them. Therefore, the intrapulmonary shunt increased in these dependent parts of the lung leading to oxygenation worsening.

### Chest wall elastance

In 22 patients with ARDS, Grasso et al. found [14] that half was responder in terms of oxygenation after a single SI and the other half was not. The explanation was that the chest wall elastance was greater in the non responders than in responders, and hence, more pressure dissipated into the chest wall and less pressure was available to distend the lung in the non responder than in the responder group. Therefore, in setting the RM what counts is not the level of the airway pressure but the level of P_L _which takes into account the chest wall elastance magnitude.

### Post-RM strategy

Lim et al. [15] investigated the effects on oxygenation of three ventilatory strategies in ARDS patients: extended sigh followed by higher PEEP than or by same PEEP as before RM, and higher PEEP alone. Oxygenation was greater in the first strategy. The authors extended this result in a comprehensive experimental study in pigs [29]. They used three ARDS models (VILI, pneumonia, oleic acid), three RMs (extended sigh, SI, pressure-controlled ventilation), and three levels of PEEP after the RM (8, 12, and 16 cm H_2_O). They found that the primary factor of the greater oxygenation was the level of PEEP after the RM. Because PEEP is an expiratory setting, it should be more relevant to tailor its level after having recruited the lung. This consideration is the background of the decremental PEEP trial, which is an attractive way to adjust PEEP [30].

## Recent advances in RM

Recently, new RMs have been described and a further assessment of their lung effects was reported that brought some additional information with clinical implications. A common feature in these new data is that they dealt with the role of time and pressure-time product during the RMs. Indeed, it has been shown that almost 80% of the recruited volume after a RM was obtained within the first 5 seconds, making the remaining 35 seconds of a 40-second RM less useful for the recruitment but potentially harmful for the lungs or the circulation [31].

In the paraquat-induced ARDS model in rats, Rzezinski et al. [32] compared a single common SI (40 cm H_2_O × 40 sec) to a progressive RM in which, starting from PEEP 15 cm H_2_O the baseline driving pressure of 10 cm H_2_O was increased by three steps of 5 cm H_2_O lasting 2 minutes each; the end-inspiratory pressure reached 40 cm H_2_O within 12 minutes and lasted 2 minutes. Lung recruitment and oxygenation were significantly greater, whereas static lung elastance, lung inflammation, alveolar epithelial cells apoptosis, and alveolar-capillary membrane injury were significantly lower with progressive RM than with the common SI. The prolongation of the RM and the pressure.time product were likely explanations for the global benefit of the prolonged RM.

Steimback et al. [33] using again the paraquat-induced ARDS model in rats, compared 180 sighs per hour, the same rate as in the early study in humans [10], set to generate Pplat of 40-cm H_2_O, to 10 sighs per hour at 20- or 40-cm H_2_O targeted Pplat, and to a common SI. The results, which are summarized in Table 2, are clearly in favour of a lower rate of sighs and a 40 cm H_2_O Pplat.

**Table 2:** Summary of the comparison of sighs in the study by Steimback et al. [33]

	SI	Sighs 180/40	Sighs 10/40	Sighs 10/20
Oxygenation	↑	↑	↓	↓
Est, L	→	↓	↓	↑
Alveolar collapse	↓	↓	↓	↑
Overdistension	→	↑	↓	→
Alveolar-capillary Membrane injury	↓	↑	↓	→
Lung apoptosis	↓	↑	↓	→
mRNA PCIII	↓	↑	↓	→

SI, sustained inflation; sighs, rate per minute/target plateau pressure; Est, L, lung static elastance; mRNAPCIII, lung expression of mRAN of procollagen III.

The arrows indicate the direction of change of each variable relative to the group of injured lungs not receiving recruitment maneuvers.

Finally, still by using the paraquat-induced ARDS in rats, Riva et al. [34] compared a common 40 cm H_2_O × 40-second SI to a RM in which the target pressure of 40 cm H_2_O was reached after 40 seconds as a ramp. Both were delivered from PEEP 0 or 5 cm H_2_O. The MR generated as a ramp from 5 cm H_2_O of PEEP reduced overdistension, alveolar collapse, lung expression of mRNA of procollagen III, and lung static elastance.

Forty patients with ARDS were randomized into SI or pressure-controlled ventilation adjusted to generate the same pressure-time product [35]. Pressure-controlled ventilation was associated with significantly greater oxygenation and with significantly less hemodynamics derangements as reflected by significantly lower central venous and pulmonary artery pressures, lower right ventricle work load, and higher cardiac output. The rapid airway pressure rising during the RM can be a factor that explains why RM can promote VILI and may worsen hemodynamics.

## Conclusions

Assessing the efficacy of RM on oxygenation only is largely insufficient and the complete evaluation, as for any ventilatory strategy in ARDS, must consider the effects on hemodynamics, lung recruitment, overdistension, stress and strain [36], and biotrauma [37]. The risks associated with RM are both at the lung level (VILI) and at the systemic level. The systemic risks that may follow RM are hemodynamics impairment or decompartimentalization of the VILI toward distant organs. The RM is a complex procedure, not standardized as yet. The factors involved in RM response largely depend on the underlying lung disease. At the present time, the previous conservative recommendations of not using RMs in routine in stable ARDS patients are still valid.

## Competing interests

The authors declare that they have no competing interests.

## Authors' contributions

CG wrote the manuscript. SD, VL, BD, FB, GB, and JCR critically reviewed the manuscript.
